# Estimation on Fixed-Bed Column Parameters of Breakthrough Behaviors for Gold Recovery by Adsorption onto Modified/Functionalized Amberlite XAD7

**DOI:** 10.3390/ijerph17186868

**Published:** 2020-09-20

**Authors:** Adina Negrea, Maria Mihailescu, Giannin Mosoarca, Mihaela Ciopec, Narcis Duteanu, Petru Negrea, Vasile Minzatu

**Affiliations:** 1 Faculty of Industrial Chemistry and Environmental Engineering, Politehnica University Timisoara, Bd. V. Parvan No. 6, 300223 Timisoara, Romania; adina.negrea@upt.ro (A.N.); mihailescumia@gmail.com (M.M.); narcis.duteanu@upt.ro (N.D.); petru.negrea@upt.ro (P.N.); 2 Research Institute for Renewable Energy of the Politehnica University Timisoara, 138 Musicescu Street, 300774 Timisoara, Romania; vasile.minzatu@student.upt.ro

**Keywords:** gold recovery, acid L-glutamic, fixed-bed column, adsorption, breakthrough curves modelling

## Abstract

The objective of this paper was to evaluate the potential of a new adsorbent material to recover Au (III) from real wastewater, in a column with a fixed bed in a dynamic regime. The material was obtained through functionalization, by impregnation of the commercial resin, Amberlite XAD 7 type, with L-glutamic acid, which has active groups –NH_2_ and –COOH. The goal of the experiments was to follow the correlation of fixed-bed column specific adsorption parameters (the effluent volume, the amounts of adsorbent, heights of the adsorbent layer in column) with the time necessary to cross the column. The experimental data obtained were modeled, using the Bohart–Adams, Yoon–Nelson Thomas and Clark models, to establish the mechanism of the Au (III) recovery process, in a dynamic regime. Also, we established the number of cycles for adsorption–desorption for which the new material can be used. We used 5% HNO_3_ (5%) as desorption agent in five adsorption–desorption cycles, until the process was no longer efficient. The degree of desorption varied between 84% and 34% from cycle 1 to cycle 5.

## 1. Introduction

Adsorption is defined as a process determined by increasing a specific compound concentration at the interface of two phases [1]. In a first stage, the compounds from the solution are transported from one phase to another and, subsequently, they adhere to the surface. The process is complex and depends on the chemistry of the surface or the sorbent and sorbate nature, as well as the nature of the solid–liquid system. Thus, adsorption of metal ions from wastewater is a cheap, efficient, non-toxic and environmentally friendly process [2,3]. 

There are two types of adsorption processes with good efficiency: adsorption in batch system and column adsorption. In the batch system, adsorption takes place between the two liquid–solid phases, where the solid phase moves continuously along with the liquid phase. The main advantages of this process are: (i) the adsorbent and the adsorbate are constantly mixed and the volume of the solution is constant; (ii) is an easy and economical method, being used in many studies. The disadvantage of this technique is that, in industry, it is not recommended to work with small amounts of adsorbent material. This makes it difficult to remove and/or recover a small amount of metal ions.

Among the adsorption processes in the column, the adsorption in a fixed bed presents some important advantages: (i) the process is continuous because the adsorbent is continuously in contact with the adsorbent; (ii) it is simple and has low costs; (iii) it can be used on an industrial scale. The disadvantages of the fixed-bed column process are: during the adsorption process, preferential routes can be formed, the adsorption being non-uniform; and the concentration profiles vary in space and time, which makes it very difficult to optimize and design the technique without a quantitative approach [4,5]. 

Gold is a precious, anticorrosive, ductile, highly malleable metal, with unique physical and chemical properties used in the electrical and electronic industry as well as in the manufacture of jewelry and coins [6,7,8]. 

Besides the adsorption process, there are a variety of Au (III) recovery methods, including: mechanical separation, hydrometallurgy, biohydrometallurgy [9,10,11,12], cementation, coagulation, solvent extraction, ion exchange, gravitational separation, flotation [8]. These methods have a number of disadvantages: (i) large amounts of sludge are generated; (ii) strongly acidic or basic solutions are used; (iii) if algae, bacteria and fungi (which have the ability to accumulate gold) are used, their multiplication must be controlled; (iv) the gold obtained has impurities requiring its purification; (v) a high cost of the operation [13,14,15,16,17]. 

Given the increasing number of environmental accidents due to the application of these gold-recovery technologies, scientists are looking for environmentally friendly technologies, such as adsorption [18]. The recovery process only makes sense if the cost of recovery is much lower than the value of the recovered gold. In addition, the restrictions imposed by waste management and strict environmental regulations require that the technological options chosen for gold recovery be economically viable and environmentally friendly. The advantage of adsorption is that gold can be recovered in small quantities from either primary or secondary solutions. Adsorption is easy to apply and if the adsorbent material is well chosen, it can have efficiencies that should not be neglected [19,20,21,22,23,24].

Regarding the recovery of Au (III), the adsorption process is the most suitable, the adsorbing properties of the material used being very important. The active groups from the adsorbent structure play an important role because they have the ability to bind by complexation, ionic bonds, hydrogen bond or electrostatic attraction. 

This paper aims to recover Au (III) from solutions generated by the galvanic coating stage within the process of obtaining printed circuit boards (PCBs) used in electronic circuits. The recovery of Au (III) in this case is achieved by adsorption in dynamic regime, in the column with a fixed bed. Thus, the aim is to establish the Au (III) recovery mechanism by adsorption in a dynamic regime, in fixed-bed column, using a newly obtained material by functionalization by the solvent impregnated resin—SIR method [25] of a cheap, commercial resin, Amberlite XAD7 with L-glutamic acid, whose pending groups are –NH_2_ and –COOH. The material is selective, relatively inexpensive, environmentally friendly, adsorbent, and was used with good results for recovering gold from dilute solutions in a batch system [26]. At the same time, the number of adsorption–desorption cycles at which the new material can be used was established.

## 2. Materials and Methods 

**Figure 1:**
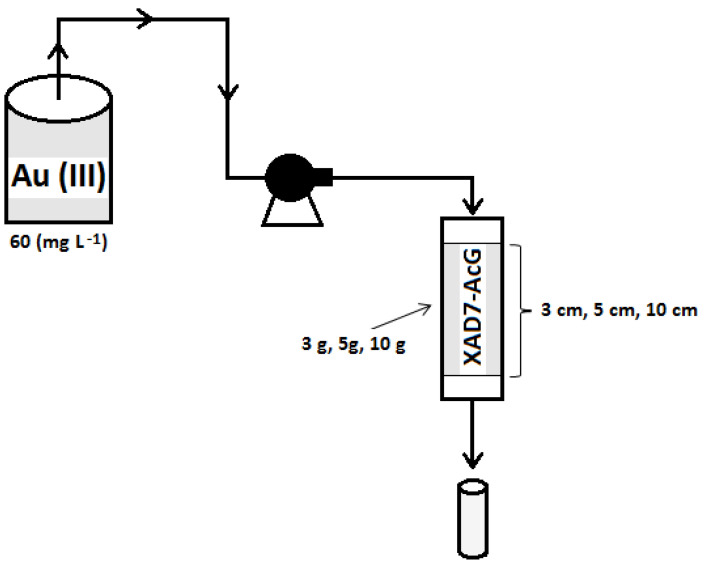
The scheme of experimental installation used for Au (III) removal in a fixed-bed adsorption column.

The surface morphology of the material obtained according to the information presented in the specialized literature [26] was studied using a scanning electron microscope Quanta FEG 250 at 500× magnitude. Adsorption experiments were realized in a dynamic regime, in a fixed-bed column. The initial concentration of Au (III) on the wastewater was 60 (mg L^−1^), being obtained by the dilution of a residual solution from cyanide baths, with an Au (III) concentration of approximately 2000 (mg L^−1^). Dilution was necessary to avoid rapid depletion of the adsorbent material, but also to highlight the fact that even in smaller quantities, Au (III) can be recovered with good efficiency. For the dynamic adsorption of Au (III) a column with a diameter of 2 cm and a length of 30 cm was used. The wastewater was pumped at the top of the column using a peristaltic pump (Heidolph SP quick) with a flow rate of 10 (mL min^−1^) and crossed in a downstream direction through the material obtained from the commercial resin of the Amberlite XAD7 type functionalized by impregnation with L-glutamic acid (XAD7-AcG). The column was loaded, sequentially, with layers of different material heights (10, 5 and 3 cm) equivalent to different masses of material (10, 5 and 3 g). Figure 1 shows the scheme of experimental installation used for Au (III) removal in the fixed-bed adsorption column. The flow in the column is kept constant and the discharge flow rate is adjusted so that its adsorption takes place as a stationary dynamic process and the adsorbent material in the column remains continuously covered with solution.

Samples of 25 mL were taken from the effluent at certain intervals. The residual concentration of Au (III) was determined by atomic absorption spectrophotometry, using a Varian SpectrAA 110 atomic absorption spectrophotometer. 

In order to determine the number of adsorption–desorption cycles at which the new adsorbent material can be used, after each adsorption cycle (for sample with amount of adsorbent 10 g and the Au (III) initial concentration of solution 60 mg g^−1^), the desorption was performed by introducing into the column as eluent HNO_3_ 5%, with a flow rate of 8 (mL min^−1^). 

## 3. Results and Discussion

### 3.1. XAD7-AcG Material Caracterization

**Figure 2:**
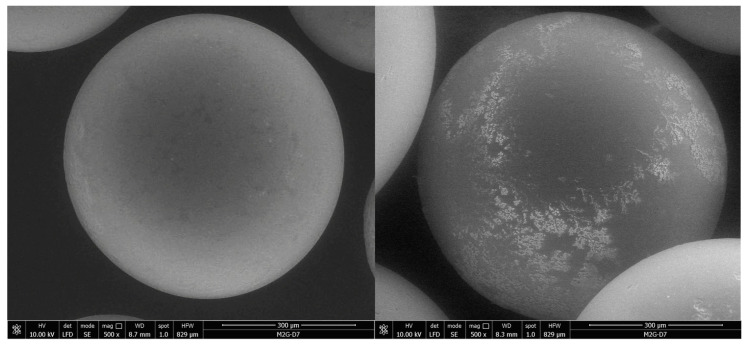
Scanning electron microscopy (SEM) for XAD 7-AcG material: (**a**) before impregnation; (**b**) after impregnation.

Figure 2 illustrate the surface morphology of the XAD7-AcG material before and after impregnation with L-glutamic acid. After impregnation, morphological changes specific to the presence of L-glutamic acid appear on the surface of Amberlite XAD7 particles.

Resin surface functionalization has also been demonstrated by other physico-chemical methods such as Fourier-transform infrared spectroscopy (FTIR) and energy-dispersive X-ray spectroscopy (EDX) in another previous study [26]. EDX reveals the presence of a small amount of nitrogen (5.83%), specific for the NH_2_ group and the FT-IR spectrum highlighted the presence of powerful vibrations specific to the Amberlite XAD7 support and L-glutamic acid respectively.

### 3.2. Fixed-Bed Column Adsorption Studies

#### 3.2.1. Mass Transfer Zone

During the adsorption process, a large number of metal ions are concentrated on the surface of the adsorbent material, respectively at the interface between the two phases, with an accumulation process taking place. The adsorption of the metal ions on the adsorbent is realized with maximum efficiency if: (i) the transport of the metal ions to the surface of the immobile layer is adequate, transport that can be realized by diffusion or dispersion; (ii) the metal ion transfer occurs at the level of the adsorbent interface; (iii) intraparticle diffusion takes place by moving the solution ions into the adsorbent pores [27].

**Figure 3:**
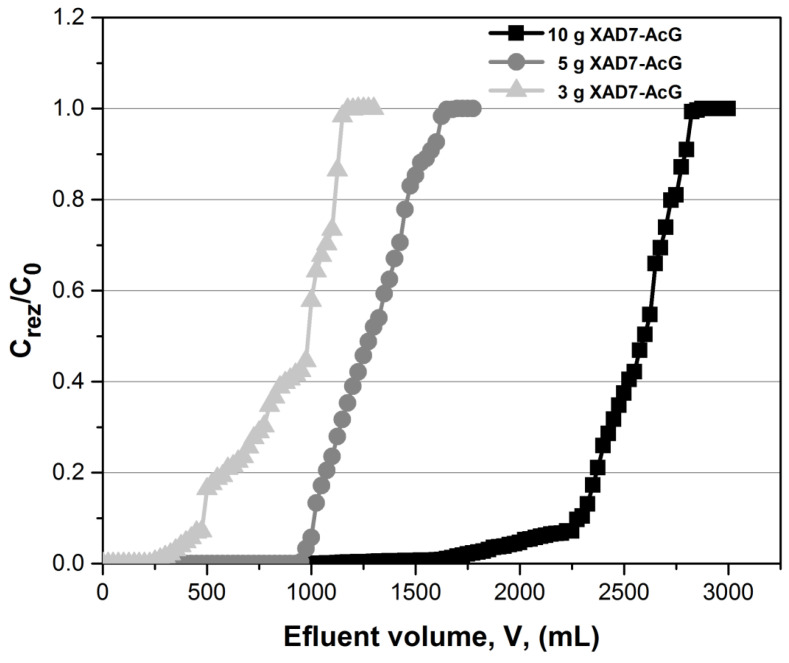
Breakthrough curves on Au (III) removal in the fixed-bed column, at various amounts of adsorbent material.

The performances of the adsorption process in the fixed-bed column are highlighted by the study of the breakthrough curves [28,29], which means following the evolution of the ratio between the residual concentration of Au (III) and its initial concentration (C_rez_/C_0_) according to the volume of effluent passed through the column, for three distinct quantities of material, respectively 3, 5 and 10 g (Figure 3).

**Figure 4:**
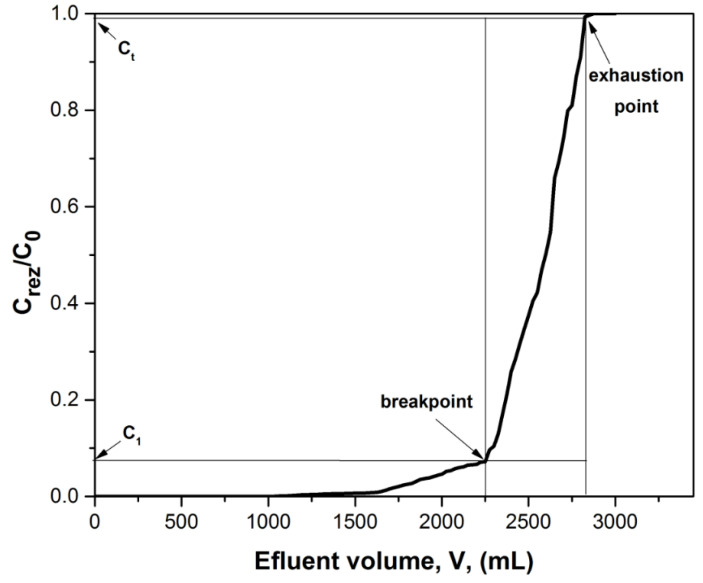
A representation of the breakthrough curve with mass transfer zone (MTZ) at an amount of 10g adsorbent material inserted in the column.

Figure 4 shows the case of the column in which 3000 mL of wastewater, with an Au (III) concentration of 60 (mg L^−1^), were passed over 10 g of XAD7-AcG material. The mass transfer zone (MTZ) is the active surface of the XAD7-AcG material where the Au (III) adsorption takes place. The waste solution Au (III) passes over a new and unused material. At the top of the column, XAD7-AcG material adsorbs Au (III) ions as soon as they come into contact; the area is called the primary sorption zone (PSZ) being delimited between the residual concentration of Au (III) at time 1 (one), C_1_, and the residual concentration of Au (III) at time t, C_t_. Thus, the first part of the liquid that is collected is without Au (III) ions, which means in this area the residual concentration of Au (III) tends towards zero. As the volume of Au (III) solution passing through the column increases, an adsorption zone begins to be defined in which mass transfer (MTZ) occurs. In this area, the adsorption process is complete, the concentration of Au (III) ions varies from the initial concentration (60 mg L^−1^) to zero, the saturation of the adsorbent material being total. This adsorption area extends over the entire height of the column depending on the contact time. The residual concentration at a given time, C_rez_, is zero and therefore the ratio C_rez_/C_0_ is zero. When the residual solution passes through the whole layer of the adsorbent material, reaching its lower part, the Au (III) ions can no longer be completely adsorbed due to the saturation of the material. This moment is called the breakpoint moment and the surface obtained corresponds to the breakpoint curve. After a while, the column is completely saturated or exhausted and the adsorption of Au (III) is no longer performed. In this case the C_rez_/C_0_ ratio is 1 (one) [30,31,32]. 

#### 3.2.2. Adsorption Models for Column Study

The important parameters for evaluating the efficiency of an adsorbent material used in a dynamic regime are: the flow of the effluent in the column, the height of the fixed layer, and the contact time [1,33]. 

In the adsorption column, phenomena of axial dispersion, external resistance of the film and resistance to intraparticle diffusion can appear. Thus, the mathematical correlation of axial dispersion, mass transfer and intraparticle diffusion is rendered by mathematical models. In order to determine the adsorption mechanism of Au (III) and to design the adsorption process in a dynamic regime it is necessary to know the evolution of the residual concentration of the effluent in time. In this case, four models can be used, namely the Adams–Bohart, Yoon–Nelson, Thomas and Clark models to analyze the breakthrough curves and for the prediction of dynamic nature of the column [34].

The Bohart–Adams Model [35] is used to describe the first part of the column breakpoint curve. The Bohart–Adams equation is linearly expressed as:

$$\ln\left(\frac{C_{t}}{C_{0}}\right)=k_{BA}C_{0}t-k_{BA}N_{0}\frac{Z}{F}$$

where: C_0_—is the influent concentration, (mg L^−1^); C_t_—is the effluent concentration, (mg L^−1^); t—is time, (min); k_BA_—is the kinetic constant of the Bohart-Adam model, (L mg^−1^ min^−1^); F—is the linear velocity calculated by dividing the flow rate by the column section area, (cm min^−1^); Z—is the bed height of column, (cm); N_0_—is the saturation concentration, (mg L^−1^).

**Figure 5:**
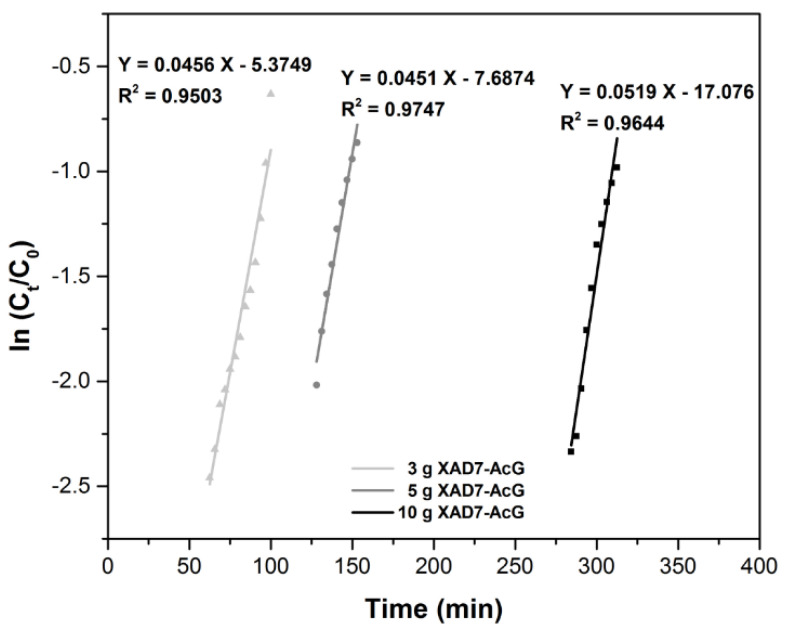
Bohart–Adams plots for the adsorption of Au (III) in the fixed-bed column, at various amounts of adsorbent material.

In Figure 5, the dependence ln (C_t_/C_0_) = f(time) was plotted. The results show that with the increase of the material layer height, respectively with the increase of the amount of material, there is a decrease of the N_0_ value, but also an increase of the k_BA_ value. The obtained regression coefficients R^2^ show that the model is not the most suitable to describe the mechanism of the dynamic adsorption process of Au (III) on the XAD7-AcG material [36].

The Yoon–Nelson Model [37] is generally adopted to describe the breakpoint curve. It is a model used especially for the single component system and does not require information about the adsorbent, such as type, physical properties or other characteristics. 

The Yoon–Nelson equation is linearly expressed as:

$$\ln\left(\frac{C_{t}}{C_{0}-C_{t}}\right)=k_{YN}t-\tau k_{YN}$$

where: C_t_—is the solution concentration at time t, (mg L^−1^); C_0_—is initial solution concentration, (mg L^−1^); k_YN_—is the rate constant, (min^−1^); τ—is the time required for 50% adsorbate breakthrough, (min).

**Figure 6:**
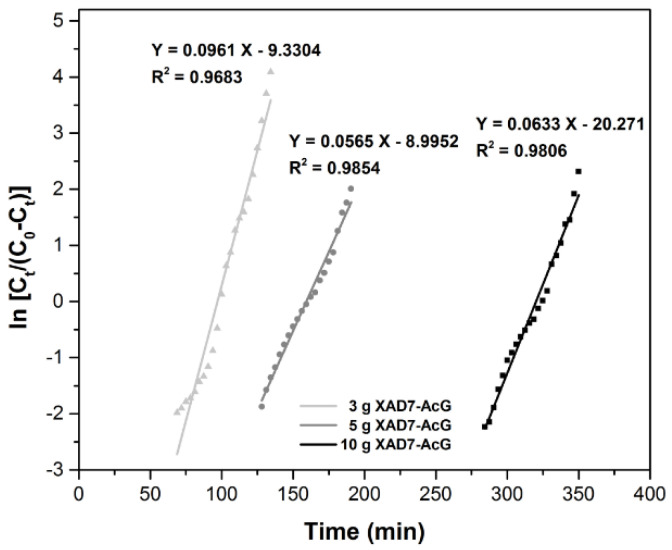
Yoon–Nelson plots for the adsorption of Au(III) in the fixed-bed column, at various amounts of adsorbent material.

The parameters τ and k_YN_ can be obtained from the plot of the function ln [C_t_/(C_0_ − C_t_)] = f(time), (Figure 6). It is observed that with the increase of the height of adsorbent layer the time required to breakthrough increases, but not in direct proportion with the adsorbent layer height. The experimental data show that the most efficient layer is the one in which 5 g of adsorbent material is used, because when the adsorbent mass doubles, τ does not become double. This fact may be due to the higher probability of preferential drainage channels occurring with the increasing amount of adsorbent material. The regression coefficient R^2^ is closer to 1, but we cannot say that the adsorption process mechanism is described in the best way by this model [34].

The Thomas model [38] is the most commonly used model to describe the adsorption column performance and to establish breakthrough curves. It is frequently used to determine the adsorption capacity of the material. The Thomas equation is linearly expressed as:

$$\ln\left(\frac{C_{0}}{C_{t}}-1\right)=\frac{k_{Th}q_{Th}m}{Q}-k_{Th}C_{0}t$$

where: C_0_—is the solution concentration in the influent, (mg L^−1^); C_t_—is the solution concentration at time t in the effluent, (mg L^−1^); k_Th_—is the Thomas rate constant, (L min^−1^ mg^−1^); q_Th_—is the equilibrium compounds uptake per g of the resin, (mg g^−1^); m—is the mass of adsorbent resin, (g); Q—is he flow rate, (mL min^−1^).

**Figure 7:**
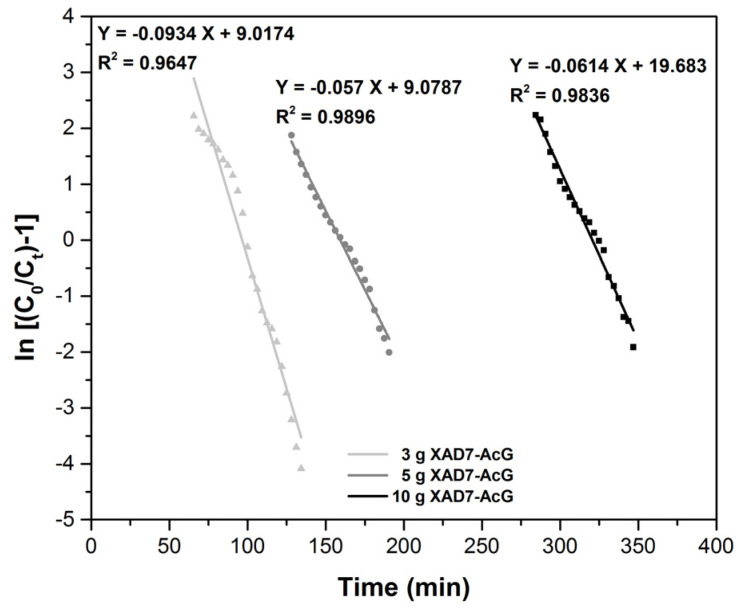
Thomas plots for the adsorption of Au (III) in the fixed-bed column, at various amounts of adsorbent material.

From the plot of ln [C_0_/(C_t_ − 1)] = f(t) are determined k_Th_ and q_Th_ (Figure 7). From the results obtained, it can be seen that the constant k_Th_ decreases as the adsorbent layer height in the column increases, due to the adsorption driving force given by the difference between the concentration of Au (III) adsorbed on the material and the Au (III) concentration in the solution [39,40,41].

At the same time, it is observed that the regression coefficient R^2^ decreases, but the values are close to 1 and the adsorption capacity has about the same value, about 13 (mg g^−1^).

Another model reported in the literature for the adsorption study in the column is the Clark model. The main assumption of this model is the use of a mass-transfer concept in combination with the Freundlich isotherm [1,42,43].

The linearized expression of the Clark model is:

$$\ln\left({\left(\frac{C_{0}}{C_{t}}\right)}^{n-1}-1\right)=\ln A-rt$$

where: C_0_—is the solution concentration in the influent, (mg L^−1^); C_t_—is the solution concentration at time t in the effluent, (mg L^−1^); n—is the Freundlich constant determined experimentally in batch; r—is the Clark model constant, (min^−1^); A—is the Clark model constant.

**Figure 8:**
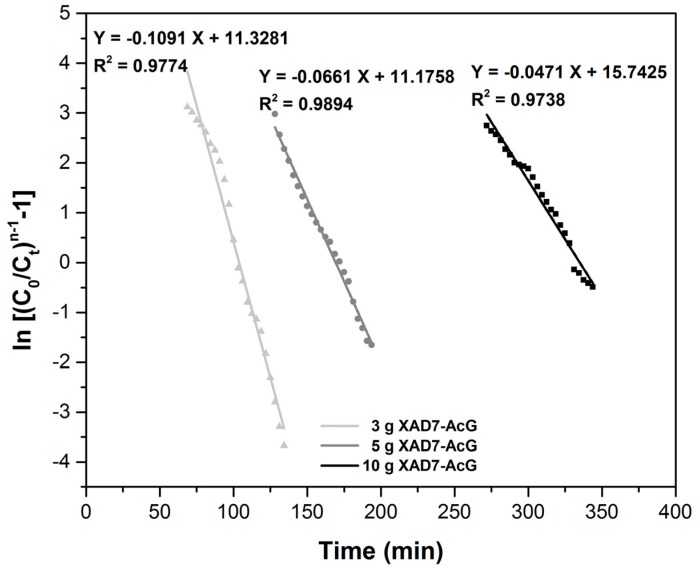
Clark plots for the adsorption of Au (III) in the fixed-bed column, at various amounts of adsorbent material.

A previous batch adsorption study showed that the Freundlich constant was n = 2.5 [26]. This value was used in the Clark model to estimate the model parameters for the Au (III) adsorption. The value of r and A parameters were evaluated by the slope and intercept of the linearized equation of the Clark model (Figure 8). The obtained correlation coefficients have good values (R^2^ > 0.97) for all the amounts of material studied. The increase of amounts of material leads to decrease of parameter r value and to increase of value of A parameter.

The difference in the models was based on the set parameters, not on matching the experimental data. Column parameters of all tested models for the adsorption process in dynamic regime are presented in Table 1. It can be observed that all the applied four models fitted to a satisfactory extent the variation in the amount of adsorbent, respectively, the variation of the material layer height. All models have shown good values of the correlation coefficient which suggests their validity in this investigation. Additionally, in the case of the Thomas model, the adsorption capacity has about the same value for all the amounts of adsorbent used, about 13 (mg g^−1^). Therefore, it can be assumed that this model best describes the mechanism of the adsorption process in a dynamic regime [41].

Maximum adsorption capacities of previously studied adsorbents for Au (III) recovery are presented in Table 2. The value of this parameter is comparable to those previously reported in the literature, even higher than other similar adsorbents.

The mechanism of Au (III) recovery by adsorption is that in most cases ionic species come into contact with the solid surface of the material with adsorbent properties. Adsorption is determined by Van der Waals forces, which are manifested between the material with adsorbent properties and the Au (III) ion. The predominant species at pH = 2 is AuCl_4_^−^. This species does not adsorb to the XAD7-AcG surface in a certain position, but moves freely, by translation, at the interface. In the aqueous solution there are three types of interaction considered competitive, namely: (i) the interaction between Au (III) ions and water; (ii) the interaction between Au (III) ions and the surface of the material with adsorbent properties and (iii) the interaction between water and the surface of the XAD7-AcG material. The efficiency of the physical adsorption process is determined by the strength of the metal ion–adsorbent surface interactions compared to the strength of the surface adsorbent–water interactions.

### 3.3. Desorption Studies

Very important for an adsorbent material is the number of adsorption–desorption cycles to which it can be subjected. The profitability, feasibility and sustainability of an adsorption process is essentially influenced by the regeneration possibilities of the adsorbent material. The desorption studies prove the practical applicability of the adsorption process, regarding the reuse of the fixed layer of adsorbent material in the column, after exhaustion [37,49]. 

**Figure 9:**
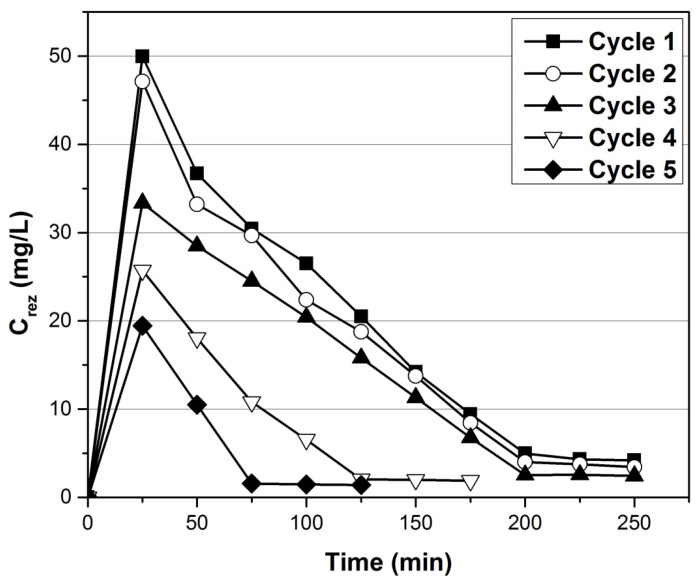
Desorption curves of Au (III) adsorbed on XAD7-AcG (10 g amounts), in five adsorption–desorption cycles.

**Figure 10:**
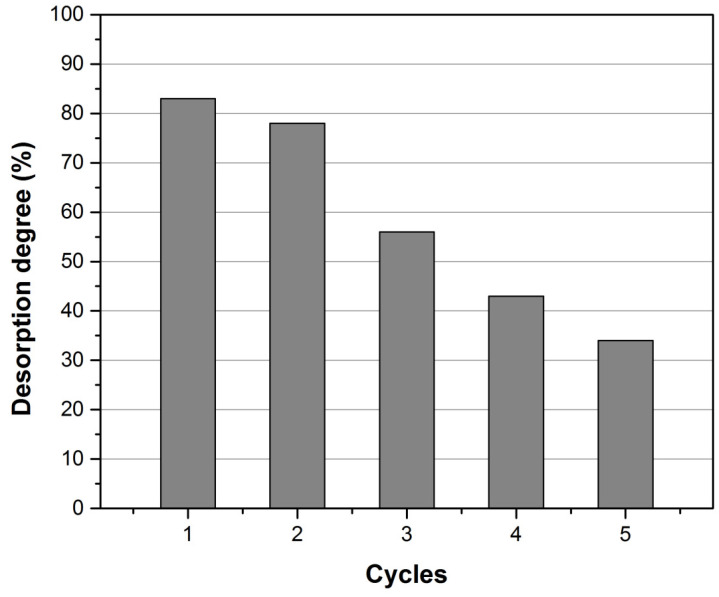
Comparative desorption degree of HNO_3_ 5%, as desorption agent, to elute Au (III) ions from XAD7-AcG adsorbent material, in consecutive cycles.

In this study, HNO_3_ 5% as desorption agent was used in five adsorption–desorption cycles. The results presented in Figure 9 and Figure 10 indicate that, with the increase of the adsorption–desorption cycles numbers there are subsequent losses in the adsorbent mass, so that the degree of desorption varies between 84% and 34% from cycle 1 to cycle 5.

Au(III) remaining on the adsorbent material, after the 5 cycles of adsorption–desorption, can be recovered in metallic form by calcination, at 873 K for 240 min, at a heating rate of 5 K/min, using controlled air oven furnaces, removing the organic part and with only the metal remaining, according to some previous studies [26].

A simple cost estimate (taking into account the price of gold on the market) led to the conclusion that for 100 g of adsorbent material, used in 5 cycles of adsorption–desorption and then calcined, the profit would be about 125 euros.

## 4. Conclusions

In this paper, the recovery of Au (III) ions from used solutions resulting from galvanic coating processes, by adsorption, in dynamic regime, using a fixed-bed column, was studied. The adsorbent was a new material obtained by impregnation of a commercial resin, Amberlite XAD7, with L-glutamic acid, which has in its structure functional groups of –NH_2_ and –COOH. In order to establish the mechanism of the dynamic adsorption process, the experimental data were modeled using the Bohart–Adams, Yoon–Nelson, Thomas and Clark models.

The Thomas model best describes the recovery process of Au (III) by adsorption in dynamic regime. The effluent flow rate, the column height, the material mass in the column and the time required to break through the column were taken into account. 

The XAD7-AcG adsorbent material can be used in five adsorption–desorption cycles, until the process is no longer efficient. The Au (III) retained in the absorbent material can be recovered by calcination, removing the organic part and leaving only the metal [14]. The adsorption capacity of the XAD7-AcG adsorbent material is large enough for the Au (III) recovery process to be cost effective.

## Figures and Tables

**Table 1:** Au (III) adsorption process parameters on fixed bed column.

Column Parameters				
**Bohart-Adams Model**	Material Amount (g)	K_B-A_ (L mg^−1^ min^−1^)	N_0_ (mg L^−1^)	R^2^
	10	8.6 × 10^−4^	1099.6	0.9644
	5	7.5 × 10^−4^	1141.9	0.9747
	3	7.4 × 10^−4^	1146.2	0.9503
**Yoon-Nelson Model**	Material Amount (g)	K_Y-N_ (min^−1^)	Τ (min)	R^2^
	10	0.0633	320.23	0.9806
	5	0.0565	159.2	0.9854
	3	0.0253	97.09	0.9683
**Thomas Model**	Material Amount (g)	K_Th_ (L mg^−1^ min^−1^)	q_Th_ (mg g^−1^)	R^2^
	10	1.02 × 10^−3^	13.376	0.9836
	5	1.05 × 10^−3^	13.379	0.9896
	3	1.56 × 10^−3^	13.520	0.9647
**Clark Model**	Material Amount (g)	r (min^−1^)	A	R^2^
	10	0.0471	68.5 × 10^−5^	0.9738
	5	0.0661	70.9 × 10^−3^	0.9894
	3	0.1091	82.4 × 10^−3^	0.9774

**Table 2:** Maximum adsorption capacities of previously studied adsorbents.

Adsorbent	Adsorption Capacity (mg g^−1^)	Reference
Sericin andalginate particle-based adsorbent SAPAS	57.91	[44]
Amberlite XAD7 type functionalized with L-glutamic acid (XAD7-AcG).	13.5	This study
Carboxymethyl chitin	11.86	[45]
Macadamia-activated carbon (MAC)	9.13	[46]
Magnetic nickeliferous pyrrhotite	6.9	[47]
Cotton cellulose	1.22 *	[48]
Activated carbon F400	0.25 **	[22]

* Original report is 6.21 (mmol/g). ** Original report is 250 (µg/g).
